# Modern Data on the Innervation of the Lophophore in *Lingula anatina* (Brachiopoda) Support the Monophyly of the Lophophorates

**DOI:** 10.1371/journal.pone.0123040

**Published:** 2015-04-22

**Authors:** Elena N. Temereva, Eugeni B. Tsitrin

**Affiliations:** 1 Department of Invertebrate Zoology, Biological Faculty, Moscow State University, Moscow, 119992, Russia; 2 Institute of Developmental Biology, Russian Academy of Sciences, Moscow, 117808, Russia; Sars International Centre for Marine Molecular Biology, NORWAY

## Abstract

Evolutionary relationships among members of the Lophophorata remain unclear. Traditionally, the Lophophorata included three phyla: Brachiopoda, Bryozoa or Ectoprocta, and Phoronida. All species in these phyla have a lophophore, which is regarded as a homologous structure of the lophophorates. Because the organization of the nervous system has been traditionally used to establish relationships among groups of animals, information on the organization of the nervous system in the lophophore of phoronids, brachiopods, and bryozoans may help clarify relationships among the lophophorates. In the current study, the innervation of the lophophore of the inarticulate brachiopod *Lingula anatina* is investigated by modern methods. The lophophore of *L*. *anatina* contains three brachial nerves: the main, accessory, and lower brachial nerves. The main brachial nerve is located at the base of the dorsal side of the brachial fold and gives rise to the cross neurite bundles, which pass through the connective tissue and connect the main and accessory brachial nerves. Nerves emanating from the accessory brachial nerve account for most of the tentacle innervation and comprise the frontal, latero-frontal, and latero-abfrontal neurite bundles. The lower brachial nerve gives rise to the abfrontal neurite bundles of the outer tentacles. Comparative analysis revealed the presence of many similar features in the organization of the lophophore nervous system in phoronids, brachiopods, and bryozoans. The main brachial nerve of *L*. *anatina* is similar to the dorsal ganglion of phoronids and the cerebral ganglion of bryozoans. The accessory brachial nerve of *L*. *anatina* is similar to the minor nerve ring of phoronids and the circumoral nerve ring of bryozoans. All lophophorates have intertentacular neurite bundles, which innervate adjacent tentacles. The presence of similar nerve elements in the lophophore of phoronids, brachiopods, and bryozoans supports the homology of the lophophore and the monophyly of the lophophorates.

## Introduction

Members of the phylum Brachiopoda are marine invertebrates with a unique body plan. Adult brachiopods are benthic animals, and most are attached to the hard substratum. Adults of the brachiopod *Lingula anatina* (Lamark, 1801) are confined to brackish intertidal habitats, where they live in burrows in the sand. Like adults of other brachiopods, *L*. *anatina* adults are suspension feeders that extract food from the surrounding water using a structure known as the lophophore. The lophophore is a specialized part of the mesosome, which bears tentacles that are covered with numerous cilia. The lophophore performs several main functions including the collecting of food particles, the brooding of embryos, and respiration. Lophophores are also known in some other bilaterians–the phoronids and bryozoans. Because of unique organization of the lophophore, which has special morphology and is supplied by special coelomic compartment, all three phyla—Brachiopoda, Phoronida, and Bryozoa or Ectoprocta—have been traditionally united in the group Lophophorata [1–4], and the lophophore has been traditionally regarded as homologous structure [5–6].

Molecular phylogenetic data indicate that bryozoans are not members of the Lophophorata and that the Lophophorata can no longer be considered as a monophyletic group [7–9]. Numerous molecular studies reveal that phoronids and brachiopods form one group, which is called the Brachiozoa, and that phoronids represent a group within inarticulate brachiopods and are referred to as brachiopods without shells [10–11]. On the other hand, some molecular data reestablish the unity of the lophophorates and support the idea that phoronids and bryozoans are closely related [12]. This idea was first suggested by Becklemishev [13], who proposed, based on characteristics of metamorphosis, that phoronids and bryozoans belong to a group that he named the Podaxonia.

Thus, the existence of the Lophophorata as a united clade remains controversial. We suggest that Lophophorata phylogeny can be clarified by the collection of detailed morphological data. The lophophore is the main morphological character used to suggest the unity of lophophorates. If the Lophophorata are truly monophyletic, we further suggest that the lophophore should have been inherited by all lophophorates from a common ancestor. If this is the case, the innervation of the lophophore in all lophophorates should possess some common features.

According to the latest morphological data, innervation of the lophophore has little in common in phoronids vs. bryozoans [14]. These findings would support the separation of bryozoans from the Lophophorata. On the other hand, the comparative analysis cannot be done without detailed descriptions of lophophore innervation in all three groups: phoronids, bryozoans, and brachiopods. Among all lophophorates, the nervous system of brachiopods has not been studied for over 100 years [15–18], and even tentacle innervation has been little studied [19–20]. Because linguliforms are a group within inarticulate brachiopods, which are the closest relatives of phoronids [11, 22–23], we expect that lophophore innervation has some common features in phoronids and brachiopods. The presence of common features of lophophore innervation in all lophophorates would help establish the homology of the lophophore and would also support the traditional view that the lophophorates represent a monophyletic group. The main aim of the research to describe the innervation of the lophophore and tentacles in *Lingula anatina* and to compare lophophore innervation in all members of the lophophorates.

## Materials and Methods

### Animals

Adults of *Lingula anatina* (Lamark, 1801) were collected from September–October 2012 in Nhatrang Bay, South China Sea, Vietnam. All investigations were made with permission and support of the Joint Vietnamese-Russian Tropical Research and Technological Centre (18 Hoang Quoc Viet Str., Caugiay Dist., Hanoi, Vietnam) and personal help of its director Academician Dmitry Pavlov and member of the Centre Prof. Temir Britaev.

The current research focused on the lophophores of *L*. *anatina*. Animals were photographed in the laboratory using a Panasonic DMC-TZ10 digital camera mounted on a binocular light microscope. Specimens were dissected at the anterior end to obtain a lophophore with mouth, tentacles, and epistome. Whole lophophores were fixed for semi-thin sectioning and scanning electron microscopy (SEM). Small parts of the lophophore were fixed for transmission electron microscopy (TEM) and confocal laser scanning microscopy (CLSM).

### Microscopy

For SEM, specimens were fixed in a 4% paraformaldehyde solution. The fixed specimens were dissected in 70% ethanol, dehydrated in ethanol followed by an acetone series, critical point dried, and then sputter coated with platinum-palladium alloy. Specimens were examined with a Jeol JSM scanning electron microscope.

For semi-thin sectioning and TEM, specimens were fixed at 4°C in 2.5% glutaraldehyde in 0.05 M cacodylate buffer containing 21 mg/ml NaCl and were then postfixed in 2% osmium tetroxide in distilled water. Postfixation was followed by en bloc staining for 2 h in 1% uranyl acetate in distilled water. The stained specimens were dehydrated in ethanol followed by an acetone series and then embedded in Spurr resin. Semi-thin and thin sections were cut with a Leica UC6 ultramicrotome. Semi-thin sections were stained with methylene blue, observed with a Zeiss Axioplan2 microscope, and photographed with an AxioCam HRm camera. Thin sections were stained with lead citrate and then examined with a JEOL JEM 100B electron microscope.

For cytochemistry, parts of *L*. *anatina* lophophores were fixed overnight in a 4% paraformaldehyde solution on a filtered of sea water and washed two times in phosphate buffer (pH 7.4) (Fisher Scientific, Pittsburgh, PA, USA) with Triton X-100 (0.3%) (Fisher Scientific) for a total of 2 h. Nonspecific binding sites were blocked with 15% normal donkey serum (Jackson ImmunoResearch, Newmarket, Suffolk, UK) in PBT overnight at 4°C. Subsequently, the larvae were transferred into primary antibody: the mixture of anti-α-Tubulin-mouse (1:700) and anti-FMRFamide-rabbit (1:800) or anti-serotonin-rabbit (1:1000) (ImmunoStar, Hudson, WI, USA) in PBT and incubated for 24 h at 4°C with gentle rotation. Specimens were washed for 24 h at 4°C in PBT and then exposed to the secondary antibody: 532-Alexa-Rabbit (1:2000) and 635-Alexa-Mouse (1:2000) (Invitrogen, Grand Island, NY, USA) in PBT for 24 h at 4°C. In the following, they were washed in PBS (three times x 60 min) and embedded in Murray Clear. In the following, they were washed in PBS (three times x 40 min), mounted on a cover glass covered with poly-L-lysine (Sigma-Aldrich, St. Louis, MO, USA), and embedded in Murray Clear. Specimens were viewed with Leica TCS SP5 confocal microscope (IDB, Moscow, Russia) and with Nikon Eclipse Ti confocal microscope (Moscow State University, Moscow, Russia). Z-projections were generated using the program Image J version 1.43. Three-dimensional reconstructions were generated using Amira version 5.2.2 software (Bitplane, Zurich, Switzerland). TEM micrographs and Z-projections were processed in Adobe Photoshop CS3 to prepare panoramas and combinations of Z-projections.

### Ethics statement

The use of brachiopods in the laboratory does not raise any ethical issues and therefore approval from regional or local research ethics committees is not required. The field studies did not involve endangered or protected species.

## Results

### Morphology of the lophophore and tentacles in *L*. *anatina*

**Fig 1:**
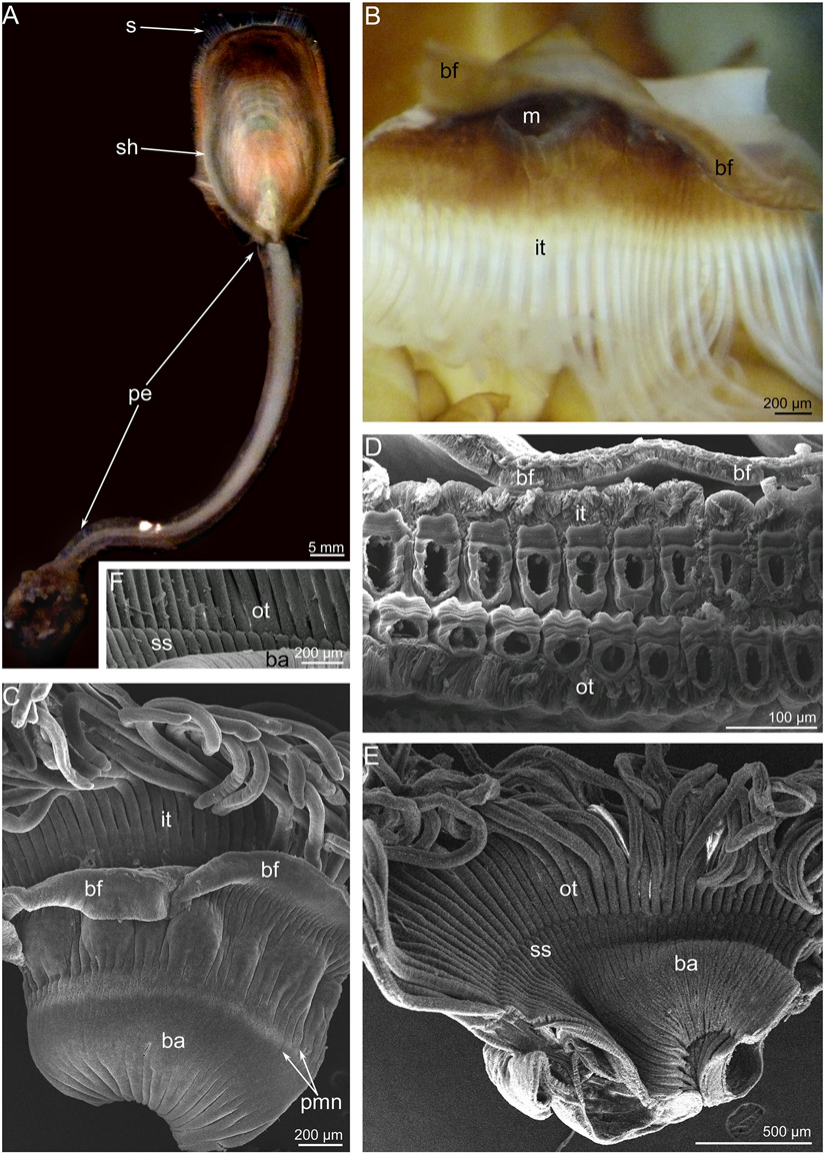
Organization of the lophophore in *Lingula anatina*. (**A**) Overview of a live *L*. *anatina*. (**B**) The central portion of the lophophore. The mouth (m) is covered by the brachial fold (bf). (**C**) A portion of the brachium, dorsal side; SEM. (**D**) Cross section of two rows of tentacles and the brachial fold (bf); SEM. (**E**) A portion of the brachium, ventral side; SEM. (**F**) Small stabs (ss) between outer tentacles. Abbreviations: ba, base of the brachium; bf, brachial fold; it, inner tentacles; m, mouth; ot, outer tentacles; pe, peduncle; s, setae; sh, shell; ss, small stubs.

**Fig 2:**
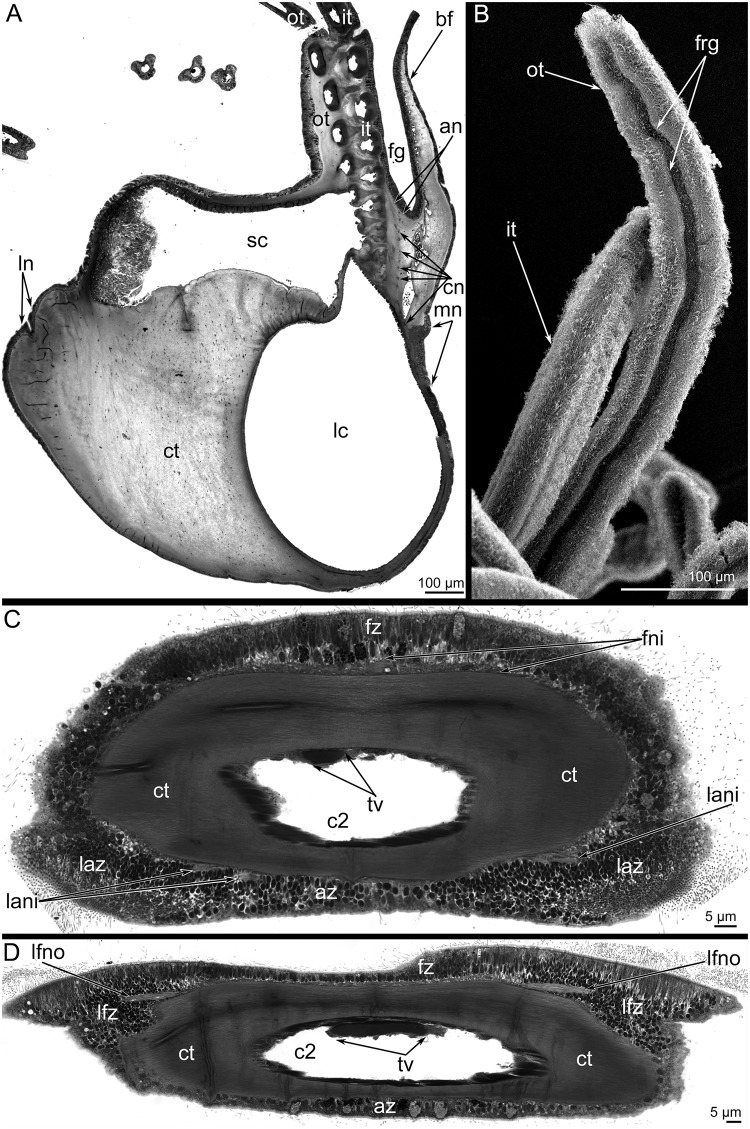
Organization of brachium and tentacles in *Lingula anatina*. (**A**) The cross semi-thin section of the brachiumn: the location of all nerve elements is shown. The dorsal side is to the right, the ventral side is to the left. (**B**) Distal potions of the outer (ot) and inner (it) tentacles; SEM. The frontal groove (frg) along the outer tentacle is clearly visible. (**C**) Cross semi-thin section of the inner tentacle. (**D**) Cross semi-thin section of the outer tentacle. Abbreviations: an, accessory brachial nerve; az, abfrontal zone; bf, brachial fold; c2, tentacular coelom; cn, cross neurite bundles; ct, connective tissue; fg, food groove; fni, frontal neurites of inner tentacle; frg, frontal groove along outer tentacle; fz, frontal zone; it, inner tentacles; lani, latero-abfrontal neurites of inner tentacle; laz, latero-abfrontal zone; lc, large canal of brachial coelom; lfz, latero-frontal zone; lfno, latero-frontal neurites of outer tentacle; ln, lower nerve; mn, main brachial nerve; ot, outer tentacle; sc, small canal of brachial coelom; tv, tentacular blood vessel.

Like many other brachiopods, *L*. *anatina* has a shell, which consists of two valves, and a long muscular peduncle that is fastened to the substratum (Fig 1A). *L*. *anatina* has a spirolophe-type lophophore. It consists of two symmetrical arms with a mouth in between. The distal ends of the brachial axes are twisted into spirals. The lophophore has a brachial fold, i.e., an epistome (Fig 1B). The brachial fold follows the lophophore and covers the tentacle bases. The food groove is located between the epistome and tentacle bases and then passes into the mouth (Fig 2A). The epistome covers the mouth from the dorsal side (Fig 1C). Tentacles are arranged in two rows: an inner row that is located near the epistome and an outer, external row (Fig 1D). Tentacles in the inner row are located in antiphase relative to the tentacles of the outer row. Between the bases of the outer tentacles are insertions, which are arranged regularly and look like small stubs (Fig 1E and 1F). Along the tentacles of both rows are several zones, which differ from each other by thickness of epidermis, density of ciliated cover, and location of glandular cells (Fig 2B–2D). There are eight zones around each tentacle: frontal, two latero-frontal, two lateral, two latero-abfrontal, and abfrontal (Fig 2C and 2D). The frontal zone faces the epistome; the abfrontal zone is opposite of the frontal zone. The shape of the tentacle in cross section differs between tentacles in the inner and outer rows (Fig 2C and 2D). Tentacles of the outer row have a deep groove, which passes along the frontal side (Fig 2B and 2D). This groove is surrounded by the thick epidermis of the latero-frontal zone. Other zones of the outer tentacles are evenly covered by cilia. Tentacles of the inner row have the thick epidermis of latero-abfrontal zones (Fig 2C). A shallow groove passes along the abfrontal zone. Frontal, lateral, and latero-frontal zones are evenly covered by cilia.

### General morphology of the L. anatina nervous system

**Fig 3:**
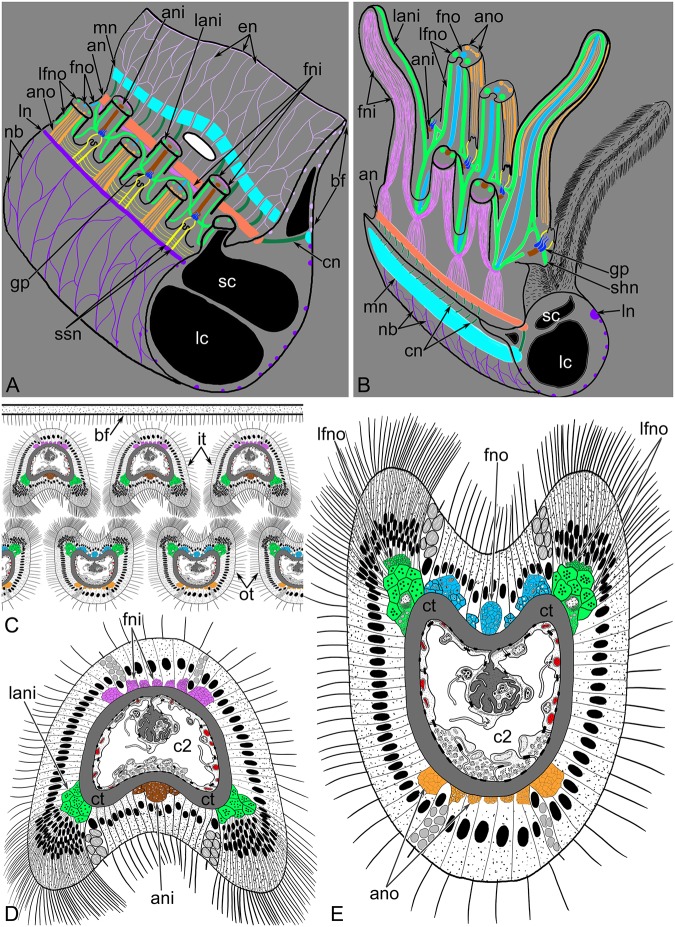
Innervation of brachia and tentacles in *Lingula anatina*; schemes. (**A**) Central portion of the lophophore with the mouth (m). Tentacles have been removed. (**B**) A portion of brachia viewed from the dorsal side. (**C**) Location of neurite bundles in the tentacles of both rows: inner and outer. (**D**) Scheme of cross section of the inner tentacle. (**E**) Scheme of cross section of the outer tentacle. Abbreviations: an, accessory brachial nerve; ani, abfrontal neurites of inner tentacles; ano, abfrontal neurites of outer tentacles; bf, brachial fold; c2, tentacular coelom; cn, cross neurite bundle; ct, connective tissue; en, neurites of the brachial fold; fni, frontal neurites of inner tentacle; fno, frontal neurites of outer tentacles; gp, group of FMRFamide-like immunoreactive perikarya; it, inner tentacles; lani, latero-abfrontal neurites of inner tentacle; lc, large canal of brachial coelom; lfno, latero-frontal neurites of outer tentacle; ln, lower nerve; mn, main brachial nerve; nb, neurites of the brachium base; ot, outer tentacle; sc, small canal of brachial coelom; shn, short nerves; ssn, neurites of small stubs; tv, tentacular blood vessel.

Three brachial neurite bundles extend along each brachium: the main brachial nerve, the accessory brachial nerve, and the lower brachial nerve. The largest neurite bundle passes along the external side of the base of the epistome (Figs 2A, 3A and 3B). It is the main brachial nerve. The main brachial nerve is connected to the accessory brachial nerve, which passes along the food groove. This connection is provided by thick cross nerves, which extend into the connective tissue of the brachial fold (Fig 3A and 3B). The accessory brachial nerve gives rise to numerous thin neurites, which pass along the inner side of the epistome. The third main nerve of the lophophore is the lower brachial nerve, which passes along the bases of the outer tentacles. The lower brachial nerve gives rise to thin circular neurite bundles, which surround each brachium and connect the lower brachial nerve and the main brachial nerve (Fig 3A and 3B).

The tentacles are innervated by accessory and lower brachial nerves. The accessory nerve gives rise to bundles of thin neurites, which pass between inner tentacles. At the tentacle base, these bundles form thick sites, where neurites subdivide into several groups (Fig 3B). The first group consists of the frontal neurites of the inner tentacles. In each inner tentacle, frontal neurites arise from two bundles: left and right (Fig 3B). The second group consists of the frontal neurites of the outer tentacles. This group of thick bundles passes from the inner to the outer side of the lophophore and then passes along the deep frontal groove of the outer tentacle. The third group of neurites gives rise to the lateral neurite bundles (Fig 3B). They pass between the two rows of tentacles. Here, each lateral neurite bundle has a T-like shape and forms two branches: one branch passes to the latero-frontal side of the outer tentacle, and the other passes to the latero-abfrontal side of the inner tentacle. The latero-frontal bundles of adjacent outer tentacles connect between tentacles and form short nerves, which are located between the two rows of tentacles along the longitudinal axis of the lophophore (Fig 3B). In the middle of each short nerve, a group of FMRF-amide-reactive perikarya is located. Each group of perikarya gives rise to the medioabfrontal neurite bundle of the inner tentacles. The outer tentacles have several abfrontal neurite bundles. They originate from the lower brachial nerve. Small stubs, which are located between the outer tentacles, are innervated by the two neurites that from a group of perikarya and pass to the lower brachial nerve (Fig 3A). Neurite bundles of the small stubs contact the abfrontal nerves of the outer tentacles via several thin, cross neurites (Fig 3A).

### Immunoreactivity and ultrastructure of the lophophore nerve elements in *L*. *anatina*

In *L*. *anatina*, all nerve elements are organized as an intraepithelial nerve plexus. This plexus forms concentrations in different places where the main nerves occur. The exception is the cross nerves, which pass in the extracellular matrix (ECM or connective tissue) and connect the main and accessory brachial nerves.

**Fig 4:**
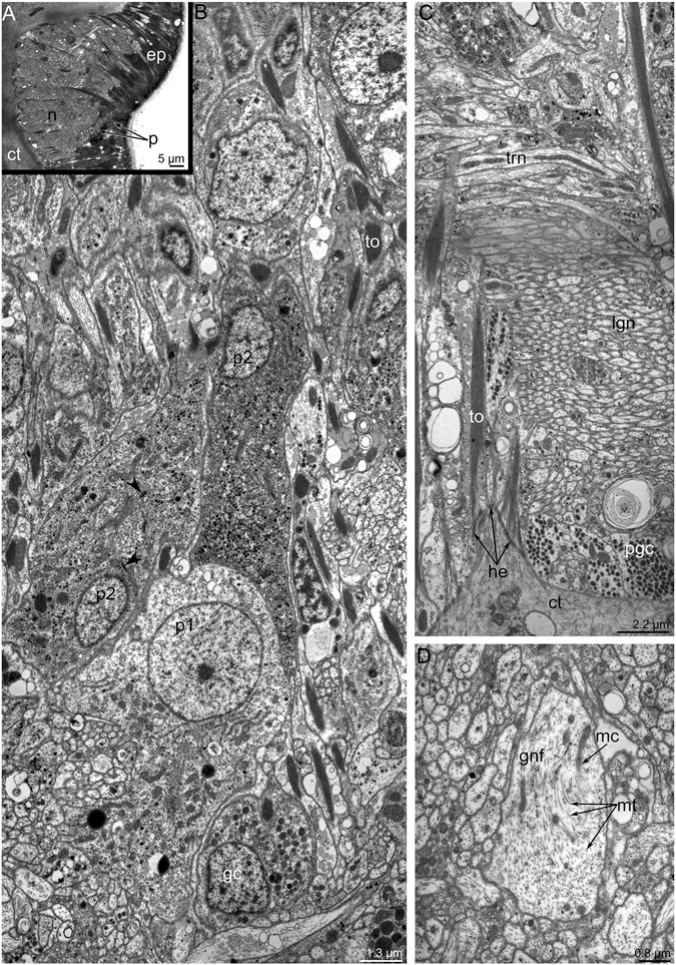
The main brachial nerve in *Lingula anatina*. (**A**) Cross semi-thin section of the main brachial nerve. (**B**) A portion of the main brachial nerve: several types of perikarya; TEM. Striated rootlets are indicated by arrowheads. (**C**) Cross section of a portion of the neuropil with neurites aligned in different directions: longitudinal (lgn) and transversal (tn) neurites; TEM. (**D**) The giant nerve fiber (gnf) in the neuropile of the main brachial nerve. Abbreviations: ct, connective tissue; ep, epidermis; gc, glial cell; he, hemidesmosome; mc, mitochondrion; mt, microtubules; n, neuropil; p, perikarya; p1, p2, perikarya of different types; pgc, projections of glial cells; to, tonofilaments.

All intraepithelial nerve elements have a similar cytological organization and stratified structure that is related to the cellular layers (Fig 4A). The internal layer contacts the basal lamina and is formed by numerous nerve fibers surrounded by processes of glial cells. The outer layer is formed by the glial cells and perikarya. All nerve elements are overarched by epidermal cells. This stratified structure is penetrated by basal projections of epidermal cells, which contain thick bundles of tonofilaments that are anchored to the basal lamina via hemidesmosomes (Fig 4B and 4C).

**Fig 5:**
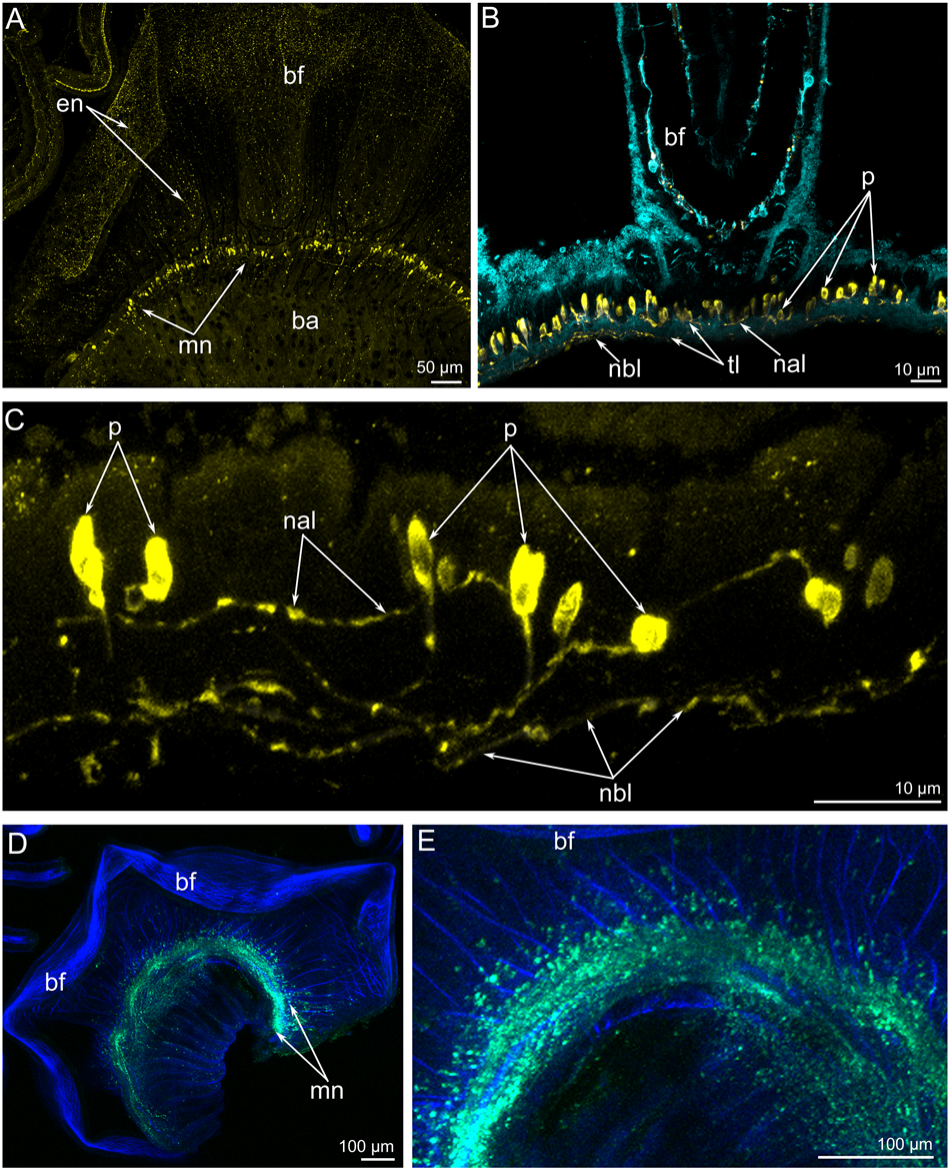
Immunoreactivity of the main brachial nerve in *Lingula anatina*. Z-projections of a portion of the brachium after mono- and double-staining for FMRF-amide (green), phalloidin (blue), α-tubulin (cyan), and serotonin (yellow). (**A**) Full Z-projection of a portion of the brachial fold (bf). (**B**) The main brachial nerve includes serotonin-like immunoreactive perikarya and a thick layer of neurites (tl), which exhibit α-tubulin-like immunoreactivity. (**C**) Several perikarya and neurites, which form apical (nal) and basal (nbl) layers. (**D**) A portion of the brachium with a thick layer of FMRFamide-like immunoreactive neurites and perikarya. (**E**) An enlargement of a portion of **D**. Abbreviations: ba, base of the brachium; mn, main brachial nerve; p, perikarya; tl, thick layer of neurites with positive α-tubulin-like immunoreactivity.

The **main brachial nerve** exhibits serotonin-like, FMRFamide-like, and α-tubulin-like immunoreactivity (Fig 5). The main brachial nerve includes many serotonin-like immunoreactive perikarya, which have different morphologies: some contact the surface of the epidermis, while others are located at the base of the epidermal cells (Fig 5A and 5C). In the main brachial nerve, serotonin-like immunoreactive neurites are few and form two thin layers: apical and basal (Fig 5C). These layers are separated by a thick layer of neurites, which exhibit α-tubulin-like immunoreactivity (Fig 5B). Serotonin-like immunoreactive neurites cross the layer of α-tubulin-like immunoreactive neurites and connect two thin layers of serotonin-like immunoreactive neurites (Fig 5C). FMRFamide-like immunoreactive neurites and perikarya form a thick layer, which is incorporated into the main brachial nerve (Fig 5D and 5E).

**Fig 6:**
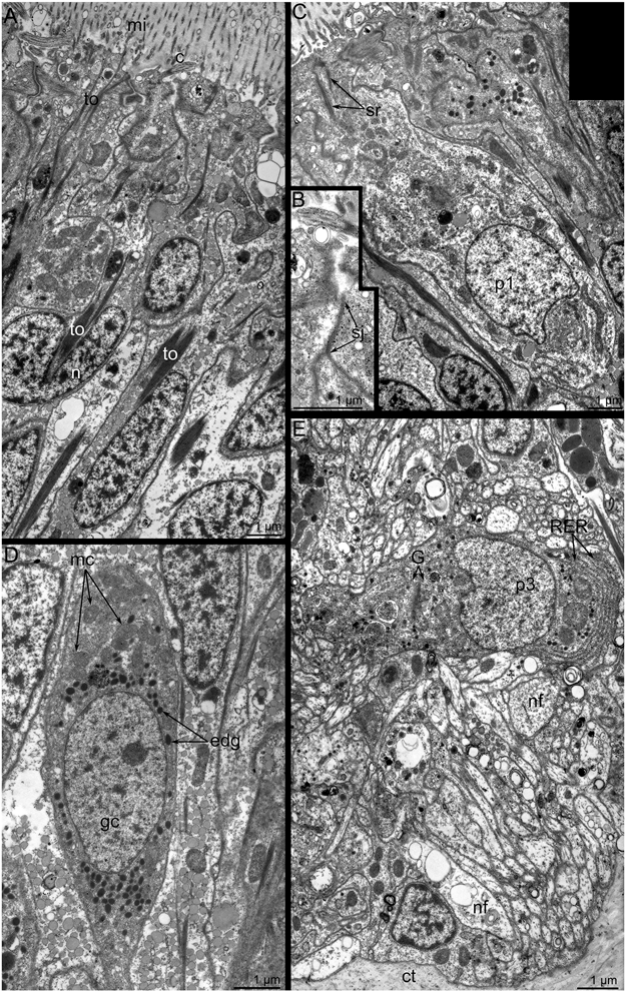
Ultrastructural details of the organization of the main brachial nerve in *Lingula anatina*, TEM. (**A**) Apical parts of epidermal cells, which bear microvilli, cilia, and tonofilaments. (**B**) Septated junction between two epidermal cells. (**C**) Apical part of a sensory cell (p1), which contacts the surface of epidermis and bears numerous synaptic vesicles. (**D**) The glial cell (gc) with numerous mitochondria (mc) and electron-dense granules (edg). Perikarya of the third type (p3) with synthetic apparatus: rough endoplasmic reticulum (RER) and Golgi complex (G). Abbreviations: c, cilium; ct, connective tissue; edg, electron dense granules; G, Golgi apparatus; gc, glial cell; mi, microvilli; mc, mitochondria; n, nucleus; nf, nerve fiber; p1, p3, perikarya of different types; RER, rough endoplasmic reticulum; sj, septate junction; sr, striated rootlet; to, tonofilaments.

Like most nerve elements, the main brachial nerve is located intraepidermally (Fig 4A) Epidermal cells, which surround the nerve elements, bear thin, long microvilli and a cilium; these cells connect to each other via desmosomes and septated junctions (Fig 6A and 6B). Epidermal cells are filled with numerous electron-dense tonofilaments, which originate in the cytoplasm of the microvilli (Fig 6A), surround the nucleus, and are anchored to the basal lamina by hemidesmosomes (Fig 4C). Cross filaments form a thick net in the apical cytoplasm. Ultrastructural study of the main brachial nerve revealed several types of perikarya (Figs 4B and 6C–6E). These perikarya differ from each other in location, density of cytoplasm, density of karyoplasm, and characteristics of vesicles, which are the dominant organelles in the cytoplasm. Some perikarya contact the surface of the epidermis and bear a cilium (Fig 6C). Perikarya of this type have electron-light cytoplasm and karyoplasm. The cytoplasm contains numerous thin, long mitochondria; short cisterns of rough endoplasmic reticulum; and a few dense-core synaptic vesicles (Fig 6C). This type of perikarya is also located within the neuropil and probably contacts the surface of the epidermis via thin apical projections (Fig 4B). Perikarya of a second type have dense cytoplasm, which is filled with numerous vesicles whose contents have a high or intermediate density. Some perikarya of this type have short, striated rootlets (Fig 4B). Perikarya of a third type have dense cytoplasm, and karyoplasm filled with dispersed chromatin. The cytoplasm of this type contains a highly developed synthetic apparatus consisting of numerous long cisterns of rough endoplasmic reticulum and Golgi apparatus (Fig 6E). The neuropil contains neurosecretory (or probably glial) cells. These cells contain numerous electron-dense granules ranging in diameter from 150 to 250 nm (Figs 4C and 6D).

A thick layer of different types of neurites is located under the perikarya. These neurites differ in diameter, orientation, and types of synaptic vesicles. Most of these neurites are 0.4 to 1 μm in diameter, are located near the basal membrane, and pass along the brachia of the lophophore. Some of neurites have the same diameter but pass in a cross direction and form an outer layer (Fig 4C). Among the smaller neurites are one to three larger neurites, with diameters from 4 to 8 μm. The cytoplasm of these giant neurites is filled with numerous thick microtubules and small mitochondria (Fig 4D).

**Fig 7:**
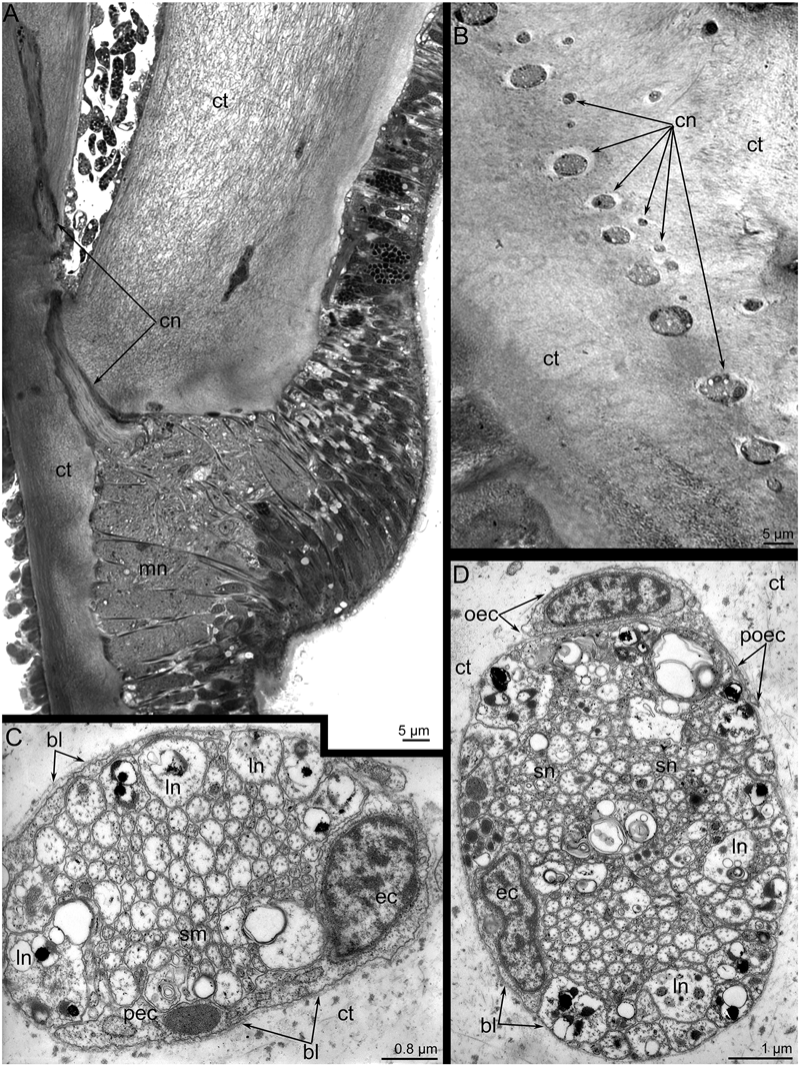
Organization of cross neurite bundles in *Lingula anatina*. (**A**) Semi-thin cross section of the main brachial nerve (mn), which gives rise to the cross neurite bundle (cn). (**B**) Semi-thin cross section of the cross neurite bundles (cn), which pass in a thick layer of connective tissue (ct). (**C**) Cross section of the cross neurite bundle, which is associated with an envelope cell (ec); TEM. This cell forms long projections (pec). Neurites of large diameter (ln) are located on the periphery, whereas neurites of small diameter (sm) occupy the central portion of the bundle. (**D**) Thin cross section of the cross neurite bundle associated with envelope cell (ec) and outer envelope cell (oec), which form thin long projections (poec). Abbreviations: bl, basal lamina; cn, cross neurite bundles; ct, connective tissue; mn, main brachial nerve.

The main brachial nerve gives rise to the **cross nerves**. These nerves originate every 10 μm from the main brachial nerve (Fig 7A). The cross nerves are not made visible by immunostaining. In the cross section of the brachium, cross nerves look like a row of spots in the ECM between the preoral coelom and the sinuses of the lophophore (Fig 7B). The diameter of these nerves ranges from 5 to 9 μm. All of these cross nerves are organized in the same way. They consist of numerous neurites of different diameter (Fig 7C and 7D). The large-diameter neurites are usually located on the periphery, whereas the small-diameter neurites occupy the central portion of the nerve (Fig 7C and 7D). The larger neurites usually contain very electron-dense inclusions. Each cross nerve is surrounded by a thick layer of basal lamina. Each nerve is also composed of an envelope cell. The envelope cell is located on the periphery of the nerve and has long thin projections that surround the neurites (Fig 7C and 7D). Cross nerves are often associated with outer envelope cells (Fig 7D).

**Fig 8:**
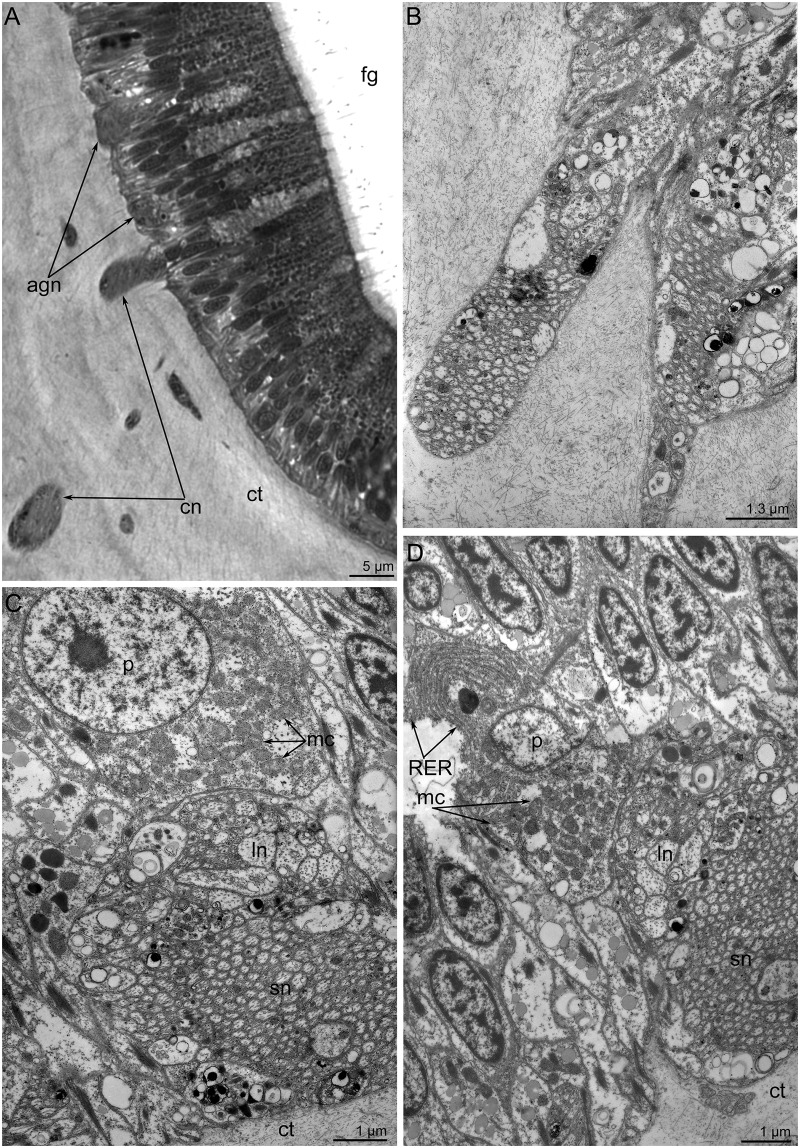
The accessory brachial nerve in *Lingula anatina*. (**A**) Semi-thin cross section of the brachium: aggregations of neurites (agn), which are associated with cross neurite bundles (cn). (**B**) A basal portion of the accessory nerve; TEM. (**C**) Aggregation of neurites of different diameter and perikarya (p); TEM. (**D**) Aggregation of neurites of small (sn) and large (ln) diameter, which are associated with perikarya (p); TEM. Abbreviations: cn, cross neurite bundles; ct, connective tissue; fg, food groove; mc, mitochondria; RER, rough endoplasmic reticulum.

In semi-thin sections, the **accessory brachial nerve** appears as a small aggregation of neurites and perikarya. This aggregation is associated with cross nerves (Fig 8A and 8B). The accessory brachial nerve consists of several groups of neurites, which are always regularly arranged: small-diameter neurites are located near the basal lamina, whereas large-diameter neurites form the upper layer (Fig 8C and 8D). A few perikarya, whose cytoplasm is filled with numerous mitochondria and synaptic vesicles, are located above the neurites (Fig 8C and 8D).

**Fig 9:**
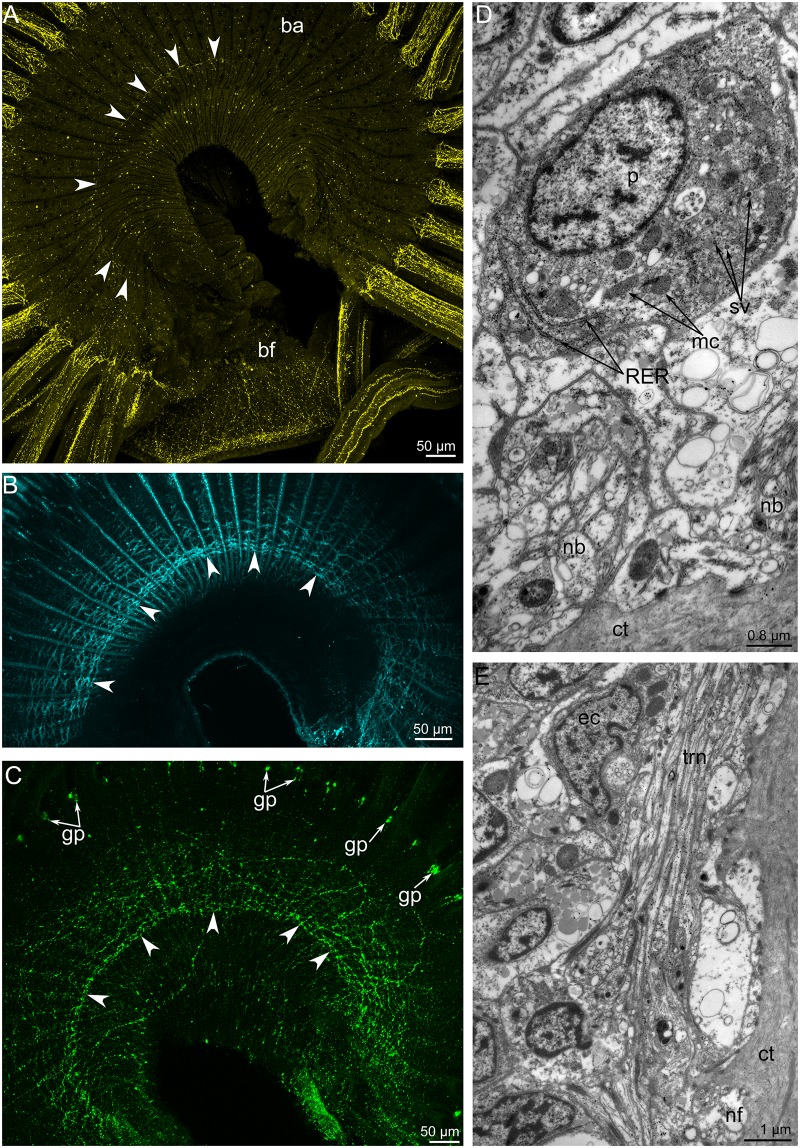
The lower brachial nerve in *Lingula anatina*. Z-projections of a portion of the brachium after staining for serotonin (yellow) (**A**), α-tubulin (cyan) (**B**), and FMRF-amide (green) (**C**). The lower brachial nerve is pointed by arrowheads. (**D**) Perikarion (p) and neurite bundles (nb) of the lower brachial nerve; TEM. (**E**) Transverse neurite bundles (trn), which pass from the tentacles and are associated with the lower brachial nerve; TEM. Abbreviations: bf, brachial fold; ct, connective tissue; ec, epidermal cell; gp, group of FMRFamide-like immunoreactive perikarya; mc, mitochondria; nf, nerve fiber; RER, rough endoplasmic reticulum; sv, synaptic vesicles.

The **lower brachial nerve** exhibits serotonin-like, FMRFamide-like, and α-tubulin-like immunoreactivity (Fig 9A–9C). It includes a few serotonin-like immunoreactive neurites (Fig 9A) and many neurites, which demonstrate FMRFamide-like and α-tubulin-like immunoreactivity (Fig 9B and 9C). According to TEM data, the lower brachial nerve consists of several large neurite bundles and a few perikarya (Fig 9D and 9E). The perikarya contain electron-dense cytoplasm and synaptic vesicles (Fig 9D). The lower brachial nerve is associated with numerous transverse neurite bundles, which pass from tentacles and extend around the brachium (Fig 9E).

**Fig 10:**
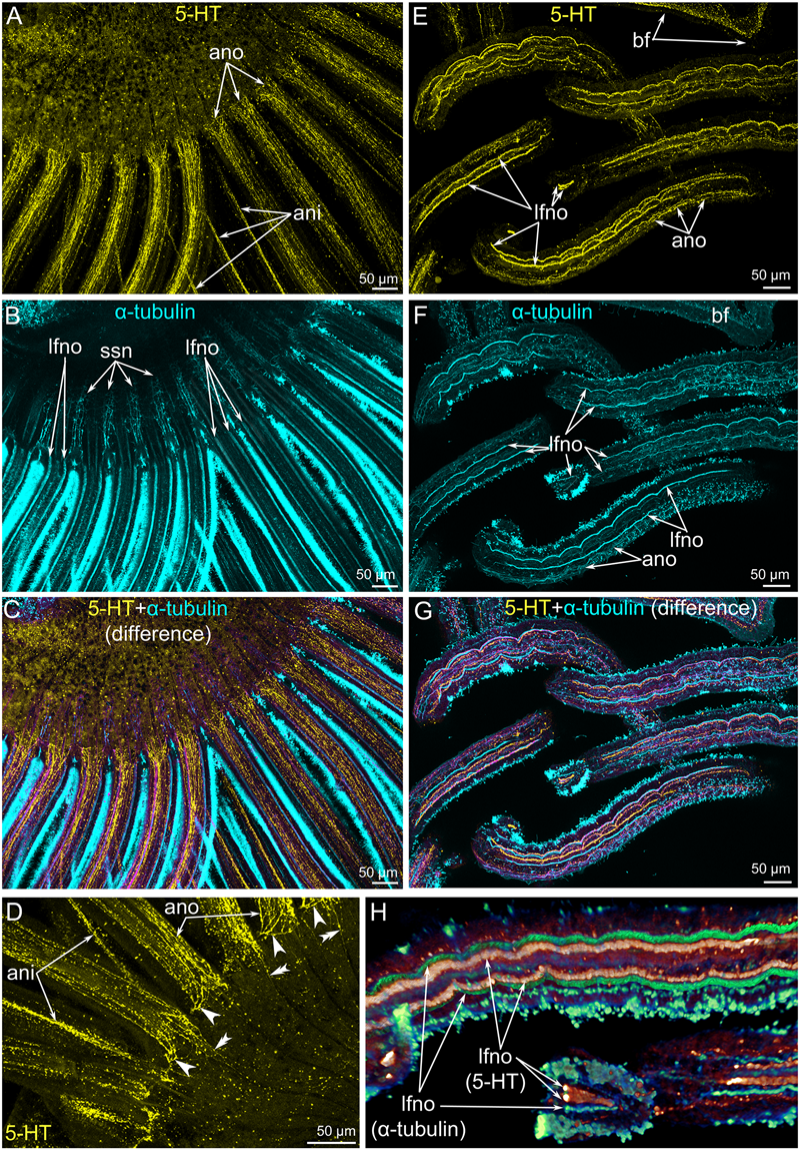
Innervation of the outer tentacles in *Lingula anatina*. Z-projections of a portion of the brachium (**A-D**) and tentacles (**E-G**) after staining for serotonin (yellow) and α-tubulin (cyan). (**A**) The tentacle bases, ventral view; serotonin-like immunoreactive elements. (**B**) The tentacle bases, ventral view; α-tubulin-like immunoreactive elements. (**C**) The tentacle bases, ventral view; **A** and **B** were combined with the Photoshop tool “Layer—Difference”. The serotonin-like and α-tubulin-like immunoreactive elements are shown in yellow and cyan (respectively) as in **A** and **B**. Elements, which exhibit both serotonin-like and α-tubulin-like immunoreactivity, are shown in magenta. (**D**) The ventral side of bases of several tentacles. Serotonin-like immunoreactive neurite bundles, which skirt the tentacle base, are indicated by arrowheads. Serotonin-like immunoreactive neurite bundles, which pass along the lophophore base, are indicated by double arrowheads. (**E**) Serotonin-like immunoreactive elements in several outer tentacles. (**F**) α-tubulin-like immunoreactive elements in several outer tentacles. (**G**) The tentacle bases, ventral view; **E** and **F** were combined with the Photoshop tool “Layer—Difference”. The serotonin-like and α-tubulin-like immunoreactive elements are shown in yellow and cyan (respectively) as in **A** and **B**. Elements, which exhibit both serotonin-like and α-tubulin-like immunoreactivity, are shown in magenta. (**H**) Three-dimensional reconstruction of serotonin-like (lfno 5-HT) and α-tubulin-like (lfno α-tubulin) immunoreactive portions of latero-frontal neurite bundles of the outer tentacles. Abbreviations: ani, abfrontal neurites of inner tentacles; ano, abfrontal neurites of outer tentacles; bf, brachial fold; lfno, latero-frontal neurites of outer tentacle; ssn, neurites of small stubs.

**Fig 11:**
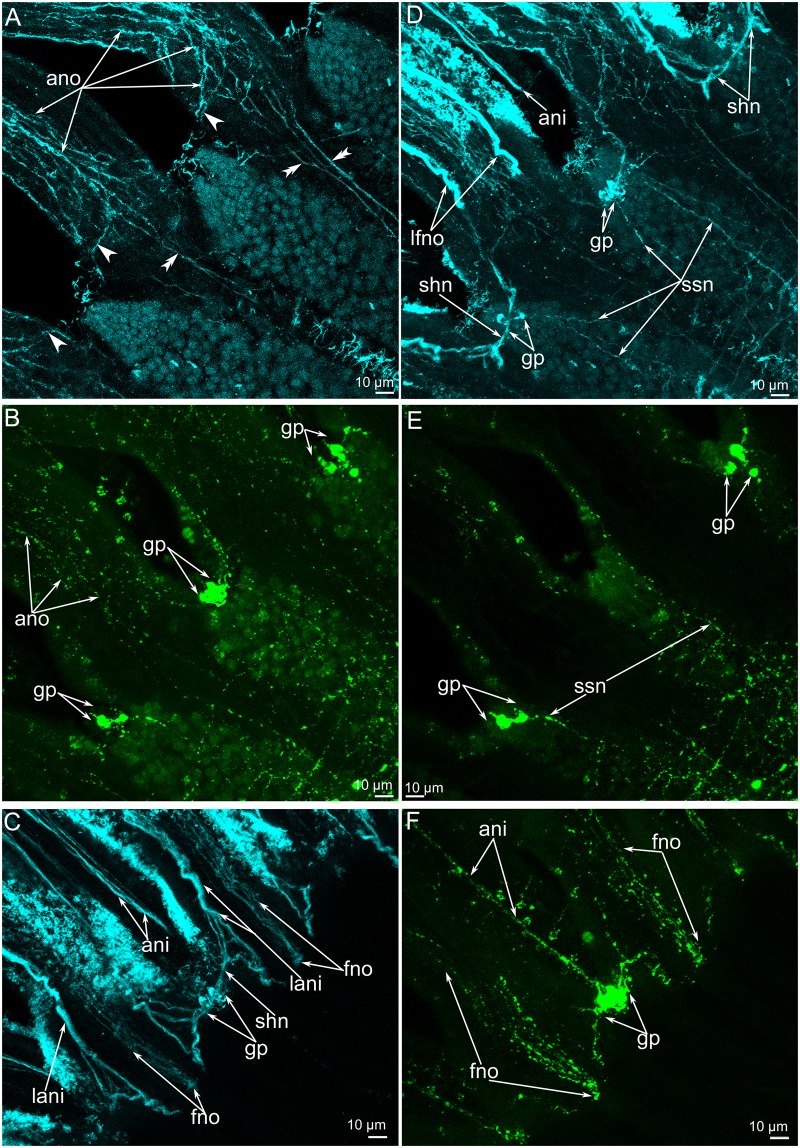
Innervation of tentacles and small stubs in *Lingula anatina*. Z-projections of a portion of the brachium after staining for α-tubulin (cyan) (**A, C, D**) and FMRFamide (green) (**B, E, F**). (**A**) Z-projection of the most ventral slides shows the nerve fiber (ano) along the abfrontal side of the outer tentacles. Some fibers skirt the tentacles (arrowheads), others pass to the brachium base (double arrowheads). (**B**) FMRFamide-like immunoreactivity in the abfrontal neurites (ano) of the outer tentacles. Groups of FMRFamide-like immunoreactive perikarya (gp) between tentacles are shown. (**C**) Z-projection of several middle slides of the stack: neurite bundles of tentacles of both rows are visible. (**D**) Z-projection of the whole stack: neurite bundles of tentacles of both rows and small stabs are visible. (**E**) Z-projection of the most ventral slides of the stack: FMRFamide-reactive nerve fibers, which pass from a group of perikarya (gp) and innervate small stabs. (**E**) Z-projection of several middle slides of the stack: the thick abfrontal neurite bundle (ani) of the inner tentacles originates from the group of FMRFamide-like reactive perikarya. Abbreviations: ani, abfrontal neurites of inner tentacles; ano, abfrontal neurites of outer tentacles; fno, frontal neurites of outer tentacles; gp, group of FMRFamide-like immunoreactive perikarya; lani, latero-abfrontal neurites of inner tentacle; lfno, latero-frontal neurites of outer tentacle; shn, short nerves; ssn, neurites of small stubs.

**Fig 12:**
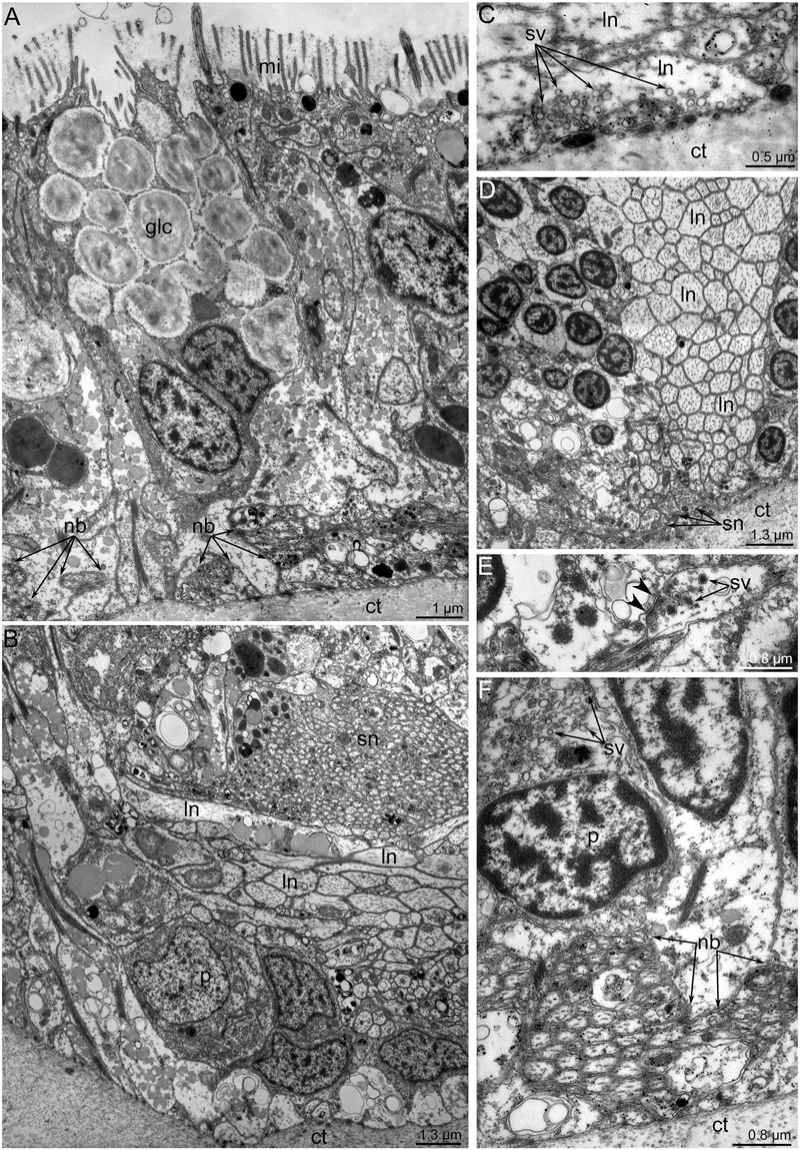
Ultrastructural data on innervation of the outer tentacles in *Lingula anatina*. Cross section of tentacles. (**A**) Abfrontal neurite bundles (nb) are associated with the gland cell (glc). (**B**) The latero-frontal neurite bundle has a complex organization and consists of perikarya (p) and neurites of large diameter (ln) and small diameter (sn). (**C**) The latero-frontal neurite bundle: a neurite of large diameter (ln), which contacts the connective tissue (ct), is filled with numerous synaptic vesicles (sv). (**D**) The latero-frontal neurite bundle is surrounded by neurites of small diameter (sn), which contact the connective tissue (ct) and contain numerous synaptic vesicles. (**E**) The presumed synapse is indicated by arrowheads. (**F**) The frontal neurite bundle is associated with perikarya (p) that contain synaptic vesicles (sv).

The **outer tentacles** are innervated by neurite bundles that extend from the lower and accessory brachial nerves. The lower brachial nerve gives rise to the abfrontal and latero-abfrontal neurites, which form a thick net along the abfrontal side of each outer tentacle (Fig 10A–10C). These neurites exhibit serotonin-like immunoreactivity and can also be recognized by staining against α-tubulin (Fig 10E–10G). At the base of each tentacle, most of the serotonin-like immunoreactive neurites form a circle, which surrounds the tentacle base (Figs 9A and 10D). A few neurites pass in the grooves between the small stubs to the lower brachial nerve (Figs 10D and 11A). The abfrontal neurite bundles exhibit weak FMRF-amide-like immunoreactivity (Fig 11B). TEM revealed several (seven to eight) abfrontal and latero-abfrontal neurite bundles, which are usually associated with gland cells (Fig 12A). The diameter of neurite bundles ranges from 1 to 3 μm. The accessory brachial nerve gives rise to the latero-frontal and mediofrontal neurite bundles (Fig 3B). The latero-frontal neurite bundles exhibit strong α-tubulin-like immunoreactivity (Fig 10B, 10C and 10F–10H) and look like thick lines along the lateral side of the tentacles. On the tip of each tentacle, the latero-frontal neurite bundles contact each other and form a loop (Fig 10F). According to TEM data, the organization of the latero-frontal neurite bundles is complex and includes numerous neurites of different types and perikarya (Fig 12B). The perikarya usually form a basal layer. The cytoplasm of the perikarya is filled with numerous electron-lucent synaptic vesicles (Fig 12B). Above the perikarya, large neurites form a second layer. Each neurite bundle contains several hundred neurites. The large neurites, whose diameters may reach 1 μm, have electron-lucent cytoplasm that is filled with thick microtubules (Fig 12B and 12D). Some of the large neurites contact the basal lamina, and their cytoplasm contains many synaptic vesicles with electron-lucent content (Fig 12C). Small neurites form a third layer and are usually located above the large neurites. The diameters of the small neurites do not exceed 0.2 μm (Fig 12B). The small neurites are located close to each other and form a compact aggregation. Some of the small neurites occur near the large neurites and in some cases contact the basal lamina (Fig 12D). Synaptic contacts are located between the neurites of the latero-frontal neurite bundle (Fig 12E). The frontal neurite bundles exhibit FMRFamide-like immunoreactivity and can also be recognized by staining against α-tubulin (Fig 11C and 11F). According to TEM data, the frontal neurite bundles are represented by several compact aggregations of neurites (Fig 12F). There are at least three large aggregations of frontal neurites: two lateral and one medial. The two lateral aggregations of neurites exhibit strong serotonin-like immunoreactivity and can be recognized by staining against serotonin (Fig 10T, 10G and 10H). The mediofrontal neurite bundle is usually associated with perikarya, which contain dense-core and electron-lucent synaptic vesicles (Fig 12G). Small stubs, which are located between the outer tentacles, are innervated from groups of FMRFamide-like immunoreactive perikarya (Figs 3A, 10B, 11D and 11E). These perikarya give rise to several thin neurites, which extend to the edge of small stubs and then extend along them (Fig 11D). These neurites demonstrate weak FRMFamide-like immunoreactivity (Fig 11E).

**Fig 13:**
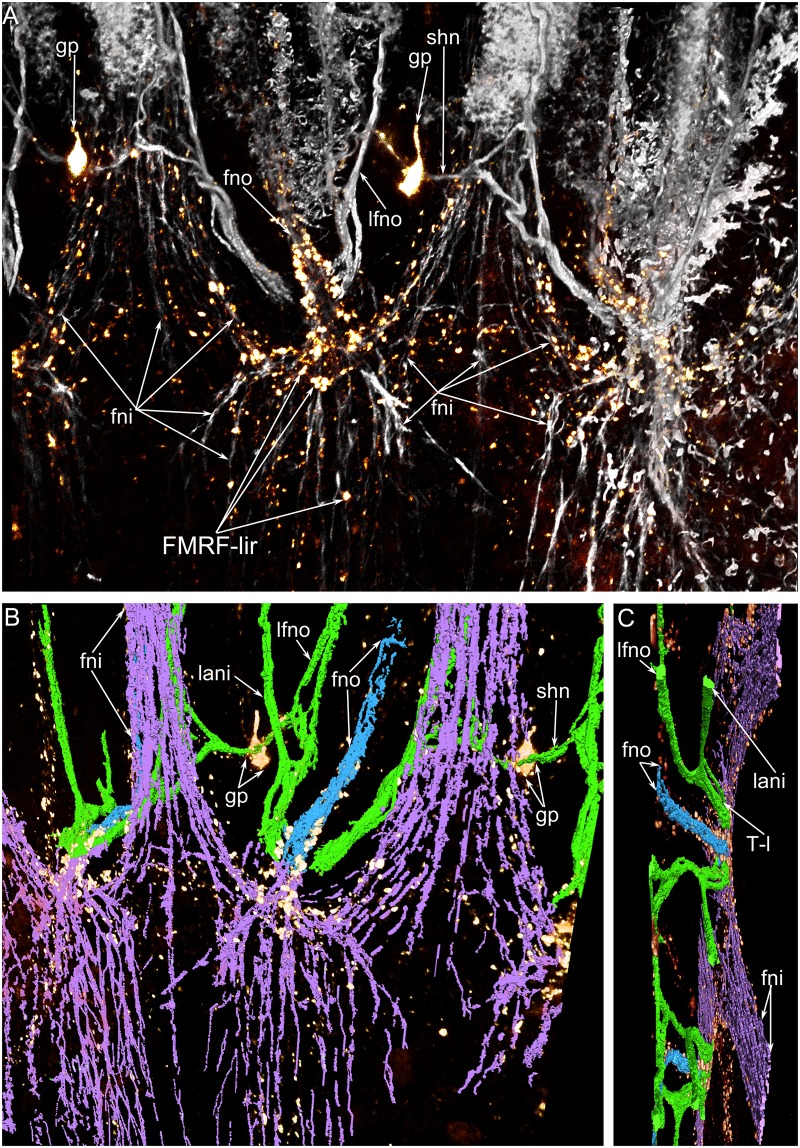
Three-dimensional reconstruction of tentacle innervation in *Lingula anatina*. (**A**) Volume rendering of α-tubulin-like (grey) and FMRFamide-like (gold) immunoreactive elements in tentacles. Reconstruction is viewed from the ventral side. Localization of nerve elements in several tentacles viewed from the ventral side. (**B**) Reconstruction of α-tubulin-like and FMRFamide-like (gold) nerve elements viewed from the dorsal side. (**C**) The same reconstruction as in (**B**) viewed from the top of the tentacles. T-like neurite bundles (T-l), which give rise to the frontal (lfno) and abfrontal (lani) neurite bundles are clearly visible. Abbreviations: FMRF-lir, FMRFamide-like immunoreactive elements; fni, frontal neurites of inner tentacle; fno, frontal neurites of outer tentacles; gp, group of FMRFamide-like immunoreactive perikarya; lani, latero-abfrontal neurites of inner tentacle; lfno, latero-frontal neurites of outer tentacle; shn, short nerves.

**Fig 14:**
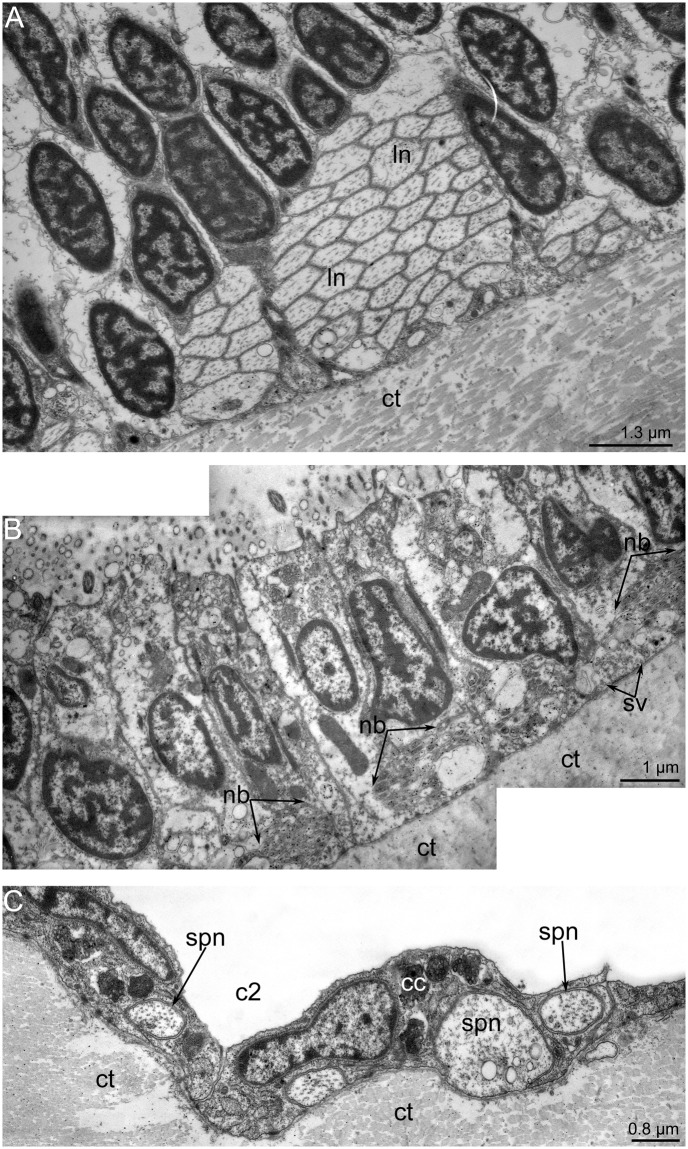
Ultrastructure of the innervation of inner tentacles in *Lingula anatina*. (**A**) The latero-abfrontal neurite bundle consists of neurites of large diameter (ln). (**B**) Several neurite bundles (nb) are located along the frontal side of inner tentacle. (**D**) Subperitoneal neurites (spn) are located between coelothelial cells (cc) of tentacles and connective tissue (ct). Abbreviations: c2, tentacular coelom; sv, synaptic vesicles.

The **inner tentacles** are innervated by the accessory brachial nerve (Fig 3A and 3B). The abfrontal side of each inner tentacle is innervated by the medioabfrontal neurite bundle, which exhibits serotonin-like, FMRFamide-like, and α-tubulin-like immunoreactivity (Figs 10A, 11C and 11F). These neurite bundles originate from the FMRFamide-like immunoreactive perikarya, which are grouped along the line between the two rows of tentacles (Fig 11F). Two thick neurite bundles extend along the latero-abfrontal sides of the tentacles (Fig 13). They are organized in the same way as the latero-frontal neurite bundles in the outer tentacles and consist of many large neurites (Fig 14A). The latero-abfrontal neurite bundles of the inner tentacles differ somewhat from the latero-frontal neurite bundles of the outer tentacles: the former are smaller, contain about 40 neurites, consist of only type of neurite, and are not associated with perikarya. The frontal side of the inner tentacles is innervated by seven to eight small neurite bundles, which exhibit serotonin-like and α-tubulin-like immunoreactivity (Figs 13 and 14B).

Tentacles of both rows contain **subperitoneal neurites**. They pass along the abfrontal and lateral sides of the tentacles and are located between the basal lamina and coelothelial cells (Fig 14C). These neurites have a large diameter (about 1 μm), electron-lucent cytoplasm, and many thick microtubules.

**Fig 15:**
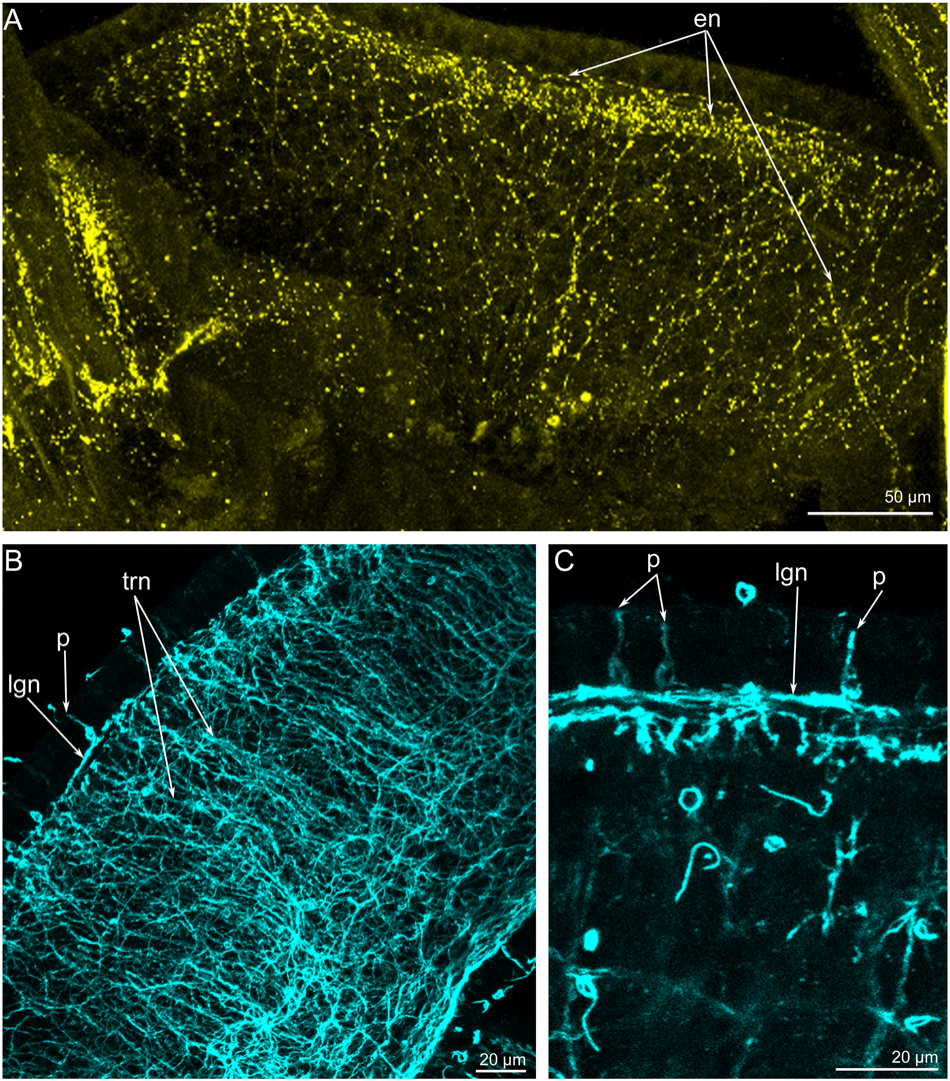
Innervation of the brachial fold and brachia in *Lingula anatina*. Z-projections of a portion of the brachial fold and brachia after staining for serotonin (yellow) (A) and α-tubulin (cyan) (B, **C**). (**A**) The brachial fold viewed from the inner (ventral) side. A thick aggregation of neurites is visible along the edge of the brachial fold. (**B**) A thick net of transversal (trn) and longitudinal (lgn) neurites on the brachium. (**C**) Portion of the surface of the brachium: in the epidermis, there are several perikarya (p), which exhibit α-tubulin-like immunoreactivity. Abbreviations: en, neurites of the brachial fold; lgn, longitudinal neurites; p, perikarya; trn, transversal neurites.

The **brachial fold** and **brachia** are innervated by numerous thin neurite bundles and a few perikarya (Fig 15). Many serotonin-like immunoreactive neurites extend along the inner and outer surface of the brachial fold. Along the edge of the brachial fold, neurites are concentrated and form a thick aggregation (Fig 15A). Each brachium is innervated by numerous neurites, which form a thick net and are mostly oriented in a circular pattern (Fig 15B). A few perikarya, which contact the surface of the epidermis, are scattered along each brachium (Fig 15C). Neurites and perikarya can be recognized by staining against α-tubulin but do not exhibit serotonin-like or FMRFamide-like immunoreactivity.

## Discussion

### Innervation of the lophophore and tentacles in brachiopods

Innervation of the lophophore in adult brachiopods has usually been studied by histological methods [15–18]. The most detailed descriptions of the brachiopod lophophore nervous system have been done for *Neocrania anomala* (= *Novocrania anomala* (Müller, 1776)), *Discinisca lamellosa* (Broderip, 1834), *Lingula anatina* (Lamarck, 1801) [17–18], and *Cryphus vitreus* (Born, 1778) [15]. According to these descriptions, the lophophore in all of these species has three main nerves, which extend along each brachium. In these earlier studies, the identity of only one of these nerves–the main brachial nerve–can be reliably established. This nerve passes along the dorsal side of the lophophore and is located at the base of the brachial fold. The main brachial nerve, which contains numerous neurites and serotonin-like and FMRFamide-like immunoreactive perikarya, is also described in the present report. According to previous reports, the main brachial nerve innervates tentacles in *L*. *anatina* [18] and contributes to the innervation of the tentacles in *N*. *anomala* [17]. In the latter case, the entire length of the main brachial nerve is connected to the accessory brachial nerve via cross nerves [17]. As shown in a previous paper [18], *L*. *anatina* lacks accessory and cross nerves, and tentacles are innervated directly by the main brachial nerve. Our study revealed the presence of both an accessory nerve and cross nerves in the *L*. *anatina* lophophore. Interestingly, the tentacles of *C*. *vitreus* are innervated by the lower brachial nerve and not by the main brachial nerve [15]. The lower brachial nerve is also found in *N*. *anomala* [17] and *L*. *anatina* [18]. In both of these species, this nerve extends along the ventral side of each brachium and corresponds to the lower brachial nerve in our report.

Thus, inarticulate brachiopods share a similar pattern of lophophore innervation in that the lophophore is served by three main longitudinal brachial nerves and numerous cross nerves.

The innervation of tentacles in brachiopods has been a little studied [19–21]. There are data about presence of mediofrontal neurite bundles along both outer and inner tentacles [19]. Although lophophore morphology differs substantially among brachiopods, the tentacles are organized similarly among the different species. In most brachiopods, there is an outer and an inner row of tentacles. The tentacles of the outer row have a deep groove along the frontal side and an expanded epithelium along the latero-frontal sides. The tentacles of the inner row have a shallow groove that passes along the abfrontal side, and the latero-abfrontal epithelium is expanded [24–26]. According to our data, the epidermis of the thickest zones is innervated by special latero-frontal (in outer tentacles) and latero-abfrontal (in inner tentacles) neurite bundles. These are huge aggregations of neurites with large diameters. In the outer tentacles, these aggregations of neurites have a complex organization and include perikarya and a layer of small-diameter neurites. Interestingly, the large-diameter neurites do not exhibit neither serotonin-like or FMRFamide-like immunoreactivity but do react strongly to anti α–tubulin. The innervation of the latero-frontal zones of the outer tentacles seems very similar to the innervation of the latero-abfrontal zones of the inner tentacles, and the nervous system of the inner tentacles appears to mirror the nervous system of the outer tentacles (Fig 3C). This similarity correlates with the density of cilia along the tentacles: along both rows, the areas along tentacles with the most cilia are innervated in the same way. Using one of the homology criteria about the special quality of the structure, we can infer that the latero-abfrontal zones of the inner tentacles and latero-frontal zones of the outer tentacles are homologous. Thus, the frontal zone is very wide in inner tentacles but narrow in outer tentacles.

According to our data, most tentacular neurite bundles in *L*. *anatina* are associated with perikarya and apparently innervate the epidermal and muscle cells of the tentacles. In *L*. *anatina*, abfrontal neurite bundles are usually associated with gland cells, which are abundant in the outer tentacles. Nerve fibers of the latero-frontal zones form synapses with epidermal cells (Fig 12E), but nerve regulation usually occurs via the ECM. For this reason, many synaptic vesicles in *L*. *anatina* are concentrated in nerve fibers, which contact the ECM of tentacles.

### Innervation of the lophophore and tentacles in lophophorates

The homology between main nerves of the lophophore in different lophophorates is difficult to establish because of differences in body plans and in the location of the main nerve centers that give rise to the main nerves of the lophophore. We did not study the location and organization of the main nerve centers, and therefore we must limit our comparisons to the general topography of the lophophoral and tentacular nerves (Table 1).

**Table 1:** Nerve elements of the lophophore and tentacles in lophophorates (see Fig 16).

Nerve elements of the lophophore and tentacles	Phoronida [29,30, 37–39]	Brachiopoda [15–21,24], herein	Bryozoa [31–36]
Nerve along the dorsal side of the lophophore	+	+	+
Nerve along the frontal side of tentacles (inner nerve)	+ small nerve ring	+ accessory nerve	+ circum-oral nerve ring
Nerve along the abfrontal side of tentacles (outer nerve)	+ tentacular nerve ring	+ lower brachial nerve	―
Innervation of frontal side of tentacles	from inner nerve	from inner nerve	from inner nerve
Innervation of latero-frontal sides of tentacles	from outer nerve	from inner nerve	from inner nerve
Presence of T-like (intertentacular) neurite bundles	+	+	+
Presence of intertentacular FMRFamide-like immunoreactive perikarya	?, in adult—, in juvenile	+	+
Presence of subperitoneal epidermal neurites in tentacles	+	+	+

“+” indicates the presence of the feature.

“―” indicates its absence.

“? “ is used if information absence.

**Fig 16:**
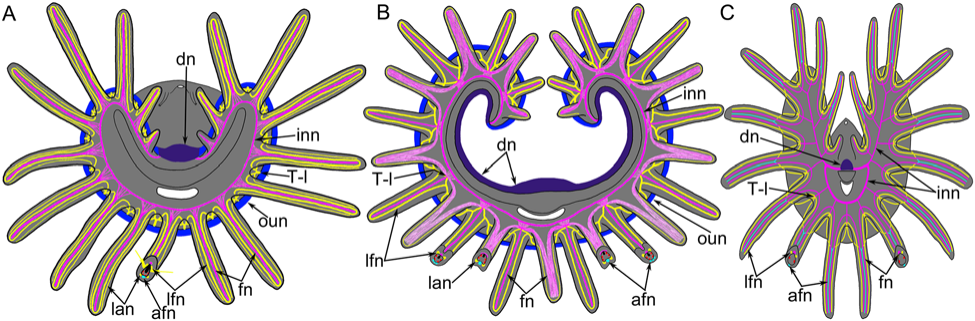
Schemes of lophophore innervation in lophophorates. The lophophore is viewed from the top; the number of tentacles is reduced; the shape of the lophophore is simplified. The same colors indicate structures that seem to be similar and may be homologous. (**A**) Phoronids. (**B**) Brachiopods. (**C**) Bryozoans. Abbreviations: afn, abfrontal neurite bundle in tentacle; dn, dorsal nerve; fn, frontal neurite bundle in tentacles; inn, inner nerve that extends along the frontal sides of tentacles; lan, latero-abfrontal neurite bundles in tentacle; lfn, latero-frontal neurite bundle in tentacle; oun, outer nerve that extends along the abfrontal sides of tentacles; T-l, T-like branches of intertentacular neurite bundles.

The lophophore is organized in many different types in all lophophorates: phoronids [27], brachiopods [28], and bryozoans [1]. At the same time, the overall plan of the lophophore is similar among all lophophorates (Fig 16). In all cases, the lophophore consists of a base and two lateral arms. The mouth is located at the lophophore base and is covered by an epidermal fold, brachial fold, or epistome. The epistome (if present) is on the dorsal side of the lophophore. The organization of the two arms of the lophophore differs among the three groups of lophophorates. The difference concerns the position of the tentacles. In phoronids and bryozoans, the tentacles surround the mouth and are located along the circle-like or horseshoe-like line. In *L*. *anatina*, the tentacles do not surround the mouth but form left and right arms, which are twisted into spirals.

All lophophorates have a dorsal nerve center, which is located on the dorsal side of the epistome base. The main brachial nerve is the dorsal nerve center in *L*. *anatina*, the dorsal ganglion in phoronids [29, 30], and the cerebral ganglion in bryozoans [31, 32]. The dorsal nerve center is very short in phoronids and bryozoans but is expanded in brachiopods (Fig 16). The compactness of the dorsal nerve center in phoronids and bryozoans correlates with lophophore organization. The tips of the arms of the lophophore are located very close to each other in phoronids and bryozoans but are located farther apart in *L*. *anatina*.

The lophophore of all lophophorates has an inner nerve. It extends along the lophophore at the frontal side of the tentacle bases (Fig 16). This inner nerve of the lophophore is the accessory brachial nerve in *L*. *anatina*, the minor nerve ring in phoronids [14], and the circum-oral nerve ring in bryozoans [32, 33].

In *L*. *anatina* and phoronids, the lophophore has an outer nerve. This nerve extends along the abfrontal side of the tentacles at their bases (Fig 16A and 16B). In *L*. *anatina*, this nerve is represented by the lower brachial nerve. In phoronids, the outer lophophoral nerve corresponds to the tentacular main nerve [29, 30]. Bryozoans lack an outer lophophoral nerve (Fig 16C). The absence of the outer nerve in bryozoans might also be attributed to a reduction due to the small body size.

In general, innervation of tentacles appears to be very similar in all lophophorates. This similarity is expressed in the presence of intertentacular neurite bundles [14, 32, 33]. In phoronids, intertentacular neurite bundles originate from the outer nerve of the lophophore [14], whereas in *L*. *anatina* and in bryozoans, they originate from the inner nerve of the lophophore [32, 33]. The intertentacular neurite bundles pass to the bases of the tentacles and branch between the tentacles in a T-like pattern, forming the latero-frontal and latero-abfrontal neurite bundles (Fig 16). Thus, in all lophophorates, the latero-frontal and latero-abfrontal neurite bundles originate from two adjacent intertentacular neurite bundles. The outer tentacles of *L*. *anatina* are exceptions to this rule: each outer tentacle in *L*. *anatina* is innervated from one intertentacular neurite bundle.

Frontal neurite bundles in *L*. *anatina* and bryozoans originate from intertentacular neurite bundles, which extend from the inner nerve of the lophophore. In phoronids, the frontal side of each tentacle is directly innervated by the inner nerve of the lophophore (Fig 16). In general, the frontal sides of tentacles are innervated by the inner nerve of the lophophore in all lophophorates.

In bryozoans, the frontal side of each tentacle is innervated by two adjacent intertentacular neurite bundles (Fig 16C). The frontal sides of inner tentacles of *L*. *anatina* are also innervated by two adjacent intertentacular neurite bundles, whereas the frontal sides of outer tentacles are innervated by one intertentacular neurite bundle (Fig 16B).

Both bryozoans and brachiopods have groups of FMRFamide-like immunoreactive perikarya at the base of the lophophore between the tentacles. The groups of perikarya between tentacles in bryozoans were first described by Gerwerzhagen [34]. FMRFamide-like immunoreactivity of these groups of perikarya is recently demonstrated by modern methods [35]. Data are lacking concerning the presence of intertentacular groups of FMRFamide-like immunoreactive perikarya in adult phoronids.

All lophophorates have subperitoneal (peritoneal) nerve fibers. In bryozoans [32,36], phoronids [37,38], and brachiopods [herein], the ultrastructure of subperitoneal cells is similar: these cells have a large diameter (about 1 μm), electron-lucent cytoplasm, and many thick microtubules. According to these features, subperitoneal neurites have much in common with the large nerve fibers of the latero-frontal and latero-abfrontal neurite bundles in tentacles of *L*. *anatina*.

### Ultrastructure of nerve elements

Little was previously known about the ultrastructure of nerve elements in brachiopods. Typically, brachiopod nerves occur as bundles of neurites and perikarya between the bases of epithelial cells [21]. According to our data, some of the nerve elements of the lophophore in *L*. *anatina* have a stratified organization, and the nerve elements form rows: the inner row consists of neurites, the middle row is formed by perikarya and the somata of glial cells, and the outer row is formed by epidermal cells. The same stratified structure of all nerve elements is described in dorsal ganglion, tentacular nerve ring, and trunk nerve plexus of phoronids [30]. In bryozoans, the nerve plexus is present in polypide and cystid [34], but does not form thick stratified net as in phoronids and brachiopods. It correlates with small size of byozoan zooids.

The ultrastructure of the main nerves is very similar in phoronids and brachiopods [30,39]. The main brachial nerve of *L*. *anatina* and dorsal ganglion of phoronids contain several types of perikarya, which differ in sets of organelles, types of synaptic vesicles, and cytoplasm and karyoplasm density. Numerous preikarya, including sensory cells that bear a cilium and contact the surface of the epidermis, are scattered in all brachial nerves of *L*. *anatina* and in the lophophoral nerves of phoronids [30,39] and bryozoans [34,35]. In phoronids [30] and brachiopods [herein], epidermal cells of the dorsal ganglion and the main brachial nerve contact each other via desmosomes and septate junctions. The presence of septate junction indicates structural strength and that small molecules are exchanged between these cells.

The giant nerve fibers have never been described in brachiopods before and are reported here for the first time. The presence of giant nerve fibers is a characteristic element of the nervous system in phoronids [29,30,39], vestimentiferans [40], and squids [41]. These fibers are usually used for fast conduction of the nerve impulses providing escape responses. In *L*. *anatina*, the giant fibers extend along each brachium and, apparently, contribute to fast conduction of the nerve impulses along lengthy brachia.

According to previous results [19–21], brachiopods contain neurite bundles in the connective tissue of the gut and tentacles [24]. In *Calloria inconspicua* (Sowerby, 1846), these neurite bundles are partially enveloped by glial cells [21,24]. In *L*. *antina*, the same bundles of nerve fibers were found in the connective tissue of the lophophore. These nerves have outer envelope cells, which are located above the thin layer of basal lamina that surrounds the neurites and glial cells.

In all lophophorates, tentacular neurite bundles are usually associated with sensory, glandular, and muscle cells. The main neurite bundles extend along the frontal, latero-frontal, and abfrontal side of each tentacle in phoronids [14,42], brachiopods [herein], and bryozoans [32]. This distribution of the neurite bundles correlates with their function, which all these tentacular zones supply. All lophophorates are upstream suspension feeders [43–46]. Lateral and latero-frontal cilia create water currents that bear food particles. Latero-frontal cilia, which have a specialized organization, are used like a sieve to catch particles [28,43,47]. The latero-frontal cells of tentacles are usually well innervated [14]. Frontal cilia generate currents from tentacle tips to the mouth. Gland cells are scattered around the tentacles [42] but are often concentrated on the abfrontal side, which is innervated by abfrontal neurite bundles.

## Conclusion

Lophophore morphology is rather similar among all three phyla of the lophophorates. The tips of the lophophore arms are located near each other in both the phoronids and bryozoans but not in brachiopods. The lophophore of all lophophorates contains identical nerve elements. Their location with respect to other organs suggests that the main brachial nerve of *L*. *anatina*, the dorsal ganglion of phoronids, and the cerebral ganglion of bryozoans are likely homologous. The accessory brachial nerve of *L*. *anatina* can be regarded as a homolog of the minor nerve ring of phoronids and the circum-oral nerve ring of bryozoans. The innervation of tentacles exhibits the following features in all lophophorates: the presence of intertentacular neurite bundles, peculiarities of the innervation of the frontal side of the tentacles, and the presence of the subperitoneal neurites. These similarities correlate in part with the similar mechanism of filter feeding, which is common in the lophophorates and may in part reflect homology between tentacles of the lophophorates. We conclude that innervation of the lophophore and tentacles has a similar ground plan in the lophophorates. Our results support the homology of the lophophore and the monophyly of the Lophophorata. This conclusion, however, should be tested with additional research that uses other methods and additional species from all three phyla of the lophophorates.
